# Etymologia: *Treponema*

**DOI:** 10.3201/eid2704.ET2704

**Published:** 2021-04

**Authors:** Fabio C. Pogliani, Rüdiger D. Ollhoff

**Affiliations:** Universidade de São Paulo, São Paulo, Brazil (F.C. Pogliani);; Pontifícia Universidade Católica do Paraná, Curitiba, Brazil (R.D. Ollhoff)

**Keywords:** etymologia, Treponema, treponemiasis, treponematosis, Treponema pallidum subsp. pallidum, bacteria, pathogenic bacteria, enzootic diseases, Fritz Richard Schaudinn, Paul Erich Hoffmann

## *Treponema* [trep′′o-ne′mə]

From the Greek *trepo* (rotate, turn) and *ne¯ma* (thread), *Treponema* is a genus of gram-negative, anaerobic or microaerophilic bacteria. They are spiral-shaped and have flagella, which extend from motors at the pole, producing undulating movement through fluids, enabling tissue invasion and dissemination (Figure). In 1905, microbiologist Fritz Richard Schaudinn and dermatologist Paul Erich Hoffmann described *Treponema pallidum* subsp. *pallidum* as *Spirochaeta pallida* from a fresh human vulvar lesion.

**Figure:**
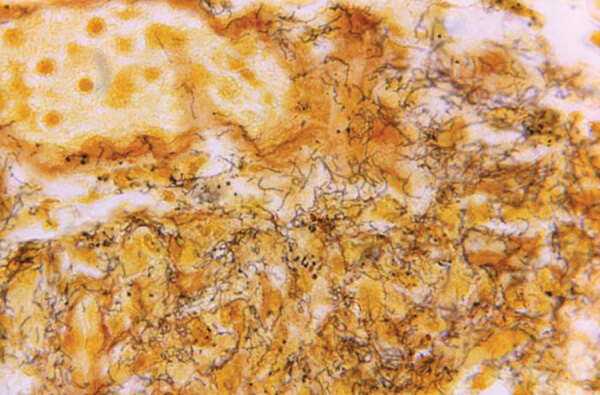
Tissue sample stained with Steiner silver stain. Image shows numerous, corkscrew-shaped, darkly-stained, Treponema pallidum spirochetes, which cause syphilis. Skip Van Orden, Centers for Disease Control, 1966.

*Treponema* spp. can invade the epidermis and oral, intestinal, and genital mucosa of humans and animals. They cause human diseases, such as syphilis, yaws, pinta, and bejel, and animal diseases, such as digital dermatitis. *T. phagedenis*, *T. pedis*, and *T. medium* infect mainly cattle. *T. paraluiscuniculi* can cause syphilis in rabbits.

Most *Treponema* spp. are not cultivable, except for *T. palllidum* subsp. *pallidum* and *T. phagedenis*. *T. pallidum* subsp. *pallidum* causative syphilis is a reemerging disease in industrialized countries. Digital dermatitis, a polytreponemal disease, is considered to be the major infectious claw disease in cattle worldwide.
